# Developing a Checklist for Cardiopulmonary Resuscitation (CPR) Quality Control in Emergency Department; a Qualitative Study

**Published:** 2019-11-02

**Authors:** Mohammad Afzalimoghaddam, Ehsan Karimialavijeh, Gholamreza Zakipour, Hadi Mirfazaelian, Amir Nejati, Pooya Payandemehr

**Affiliations:** 1 Emergency Department, Imam Khomeini Hospital Complex, Tehran University of Medical Sciences, Tehran, Iran.; 2 Emergency Department, Emergency Medicine Research Center, Tehran University of Medical Sciences, Tehran, Iran.; 3 Emergency Department, Shariati Hospital, Tehran University of Medical Sciences, Tehran, Iran.; 4 Emergency Department, Sina Hospital, Tehran University of Medical Sciences, Tehran, Iran.; 5 Prehospital and hospital emergency research center, Tehran University of Medical Sciences, Tehran, Iran.

**Keywords:** Cardiopulmonary Resuscitation, Heart arrest, quality control, Emergency Medicine

## Abstract

**Introduction:**

Monitoring the quality of cardiopulmonary resuscitation (CPR) could help in achieving favorable outcomes, decreasing mortality, and preventing post-CPR neurologic sequels. This study aimed to generate a user-friendly checklist for CPR quality control in emergency department (ED).

**Method:**

A qualitative study was performed between January and December 2018. In the first step, two emergency medicine specialists searched currently available databases and extracted the factors related to CPR quality. Afterward, two sessions of focus group discussions were held. The participants included four emergency medicine specialists, two ED managers, one anesthesiologist, and one cardiologist. Subsequently, 20 medical specialists, consisting of 10 emergency medicine specialists, six anesthesiologists, and four cardiologists, were invited to a Delphi panel in order to rate the extracted items from the prior group discussions.

**Results:**

During the two rounds of focus group discussions, 38 items related to the quality of CPR were identified. A Delphi panel evaluated the items; 31 items with at least 75% agreement were selected. These 31 items were included in the final checklist and after a pilot study and adjustment of its content they were sorted in 10 categories as follows: 1. chest compression, 2. airway, 3. bag-mask ventilation, 4. cardiac monitoring, 5. defibrillation, 6. intravenous (IV) drug delivery, 7. Medications, 8. Advanced airway, 9. CPR sequence, and 10. Reversible causes.

**Conclusion:**

Our study provides a checklist for monitoring the quality of CPR in ED, but it is still necessary to include other factors related to the ED environment on this checklist.

## Introduction

Monitoring the quality of cardiopulmonary resuscitation (CPR) in the emergency department (ED) is an essential requirement for achieving favorable outcomes, decreasing mortality, and preventing post-CPR neurologic sequels (1). Despite continuous training and education, even trained healthcare providers may perform suboptimal CPR (2). According to the CPR quality consensus statement released by the American Heart Association (AHA), CPR quality should be monitored during all resuscitations (3). The relevant metrics include parameters such as chest compression rate and depth, ventilation rate, compression pause duration, and incomplete chest recoil (4). 

The quality of CPR has many aspects including team management, patient management, utilizing standard medical devices (cardiac monitors, electroshock devices, etc.) and performing the process of CPR based on the latest guidelines recommended by the AHA (5).A system for measuring the quality of CPR should be part of any hospital’s safety protocol. In order to control the quality of CPR in the ED, investigators need to use a checklist containing all aspects of high-quality CPR. Furthermore, ED has different specifications compared with other parts of the hospital. Overcrowding, lack of resources and teamwork have a negative impact on medical care in EDs, including CPR (6). Based on above-mentioned points, the present study aimed to invite a group of medical specialists in the field of resuscitation to review the current resuscitation guidelines in the literature and generate a user-friendly checklist for quality control of CPR in the ED.

## Methods

***Study design and setting***

This was a qualitative study performed between January and December 2018. The current literature was searched for preferred items regarding CPR quality control; then using the focus discussion and Delphi panel, a CPR quality control checklist was developed. The local Ethics committee of the TUMS approved the conduct of the study, and all patient data were protected with respect to patients’ privacy (Ethics code: 42851300297.).

 ***Data gathering***

In the first step, two emergency medicine specialists searched currently available databases (Ovid, PubMed, Medscape, Google Scholar, Web of Science, and Scopus) and extracted the factors related to CPR quality (Table 1). We used a broad search strategy to identify studies assessing the quality of CPR in emergency department. The search strategy included Medical Subject Headings and keywords related to "cardiopulmonary resuscitation", "heart arrest", "quality control", "and emergency medicine".

Afterwards, two sessions of focus group discussions were held. The duration of each session was two hours. A group of medical specialists, consisting of four emergency medicine specialists, two ED managers, one anesthesiologist, and one cardiologist, participated in the focus group discussions.

**Table 1:** Factors related to high-quality cardiopulmonary resuscitation (CPR) in the literature

**Factor** **s**
Post CPR survival rate before hospital discharge
Rate of return of spontaneous circulation (ROSC)
Chest compression depth
Chest compression rate
Compression-ventilation ratio
CPR duration
Delay to initiate CPR
Initial cardiac rhythm
Brain function after ROSC

**Table 2:** Items (checklist) for monitoring cardiopulmonary resuscitation (CPR) quality

**Category/Items**	Agreement*
**Chest compression**	
Put hands on the lower third of the sternum in the midline. Compression depth at least 2 inches for adults. Allow complete chest recoil after each compression. Compression rate 100-120/minute. Change compressor after 2 minutes with minimum delay.	0.8 1 1 1 0.95
**Airway**	
Open airway properly.	0.9
**Bag-mask Ventilation **	
Proper mask seal. Proper mask holding by the rescuer. Keep compression-ventilation ratio at 30:2. Insert oral airway. Deliver each breath in one second.	0.78 0.82 1 0.76 0.86
**Cardiac monitoring**	
Initiate cardiac monitoring immediately. Proper diagnosis of cardiac rhythm. Proper pulse check in 10 seconds.	0.78 0.9 0.92
**Defibrillation**	
Set shock energy properly. Put paddles in proper position. Insert sufficient pressure on paddles. Warn the CPR team to be clear of the patient before shock delivery. Continue the massage immediately after shock delivery.	0.96 0.9 0.77 1 1
**IV drug delivery**	
Deliver drugs at a proper time based on the cardiac rhythm. Provide IV access (peripheral, central, intra-osseous, and cutdown).	0.98 0.95
**Medications**	
Choose drugs based on correct indications. Proper drug dosage.	1 0.95
**Advanced airway**	
Provide advanced airway. Minimum delay in cardiac massage when inserting the airway. Correctly confirm the location of the airway. Proper ventilation rate and volume. Use 100% oxygen.	0.96 1 1 0.96 0.8
**CPR sequence**	
Stick to the proper sequence of the AHA Algorithms.	1
**Reversible Causes**	
Consider reversible causes. Use proper interventions to resolve reversible causes.	0.97 0.95

*Based on Cohen’s kappa coefficient. IV: intravenous; AHA: American Heart Association.

**Figure 1:**
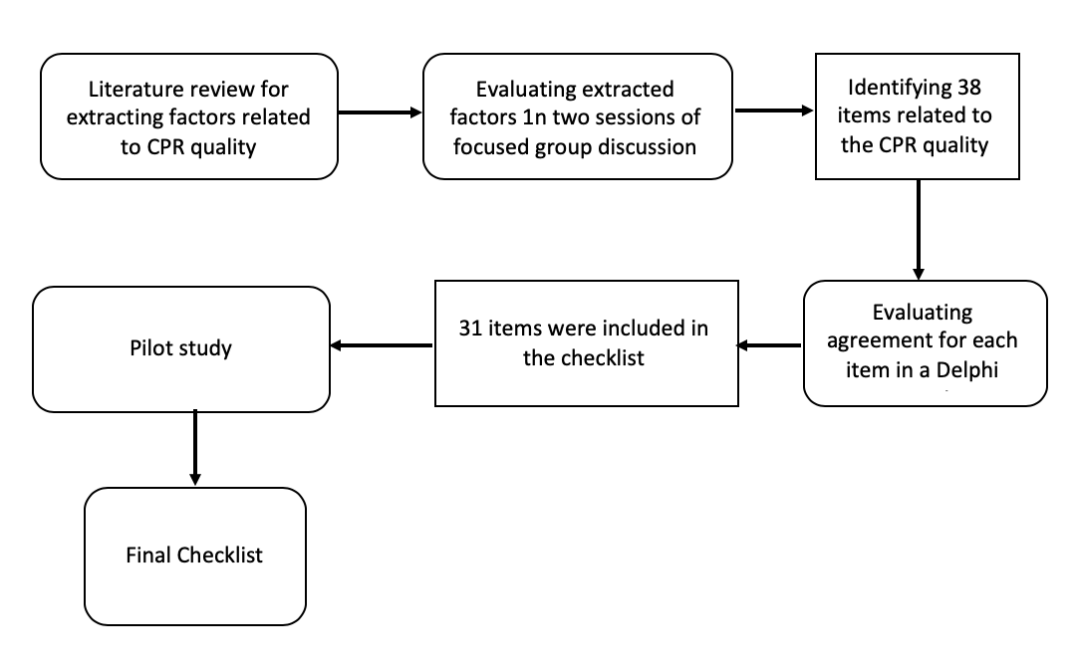
The flow chart of the study

The participants were all faculty members of Tehran University of Medical Sciences (TUMS) with at least five years of professional experience in the field of emergency medicine and resuscitation or ED management. 

The discussion aimed to reach at least a 90% consensus about each extracted factor. Subsequently, another 20 medical specialists, consisting of 10 emergency medicine specialists, six anesthesiologists, and four cardiologists, were invited to a Delphi panel. They rated the items derived from the prior focus group discussions in order to reach at least 75% agreement for each item. A Likert scale (between 0 for strongly disagree and 5 for strongly agree) was used to rate the items.

These items were selected to generate a checklist for quality control of CPR in ED. This checklist was piloted in a university-affiliated urban ED for three months assessing 70 cardiac arrest patients and adjustments were made by a team of medical specialists, including four emergency medicine specialists, one cardiologist, and one anesthesiologist. Eventually, a checklist for quality control of CPR in ED was prepared. 

***Statistical analysis***

The inter-rater agreement for each item among medical specialists was analyzed using the kappa statistic (κ), where κ greater than 0.75 was considered as complete agreement (7). Data analysis was performed using SPSS (Version 22, SPSS Inc., Chicago, IL). 

## Results

The flow chart of the study is depicted in Figure 1. After a literature review by two emergency medicine specialists, factors related to CPR quality were extracted and summarized into nine categories (Table 1).  

During two rounds of focus group discussions, 38 items related to the quality of CPR were identified in order to formulate a checklist. The items on this primary checklist were evaluated by a Delphi panel; 31 items with at least 75% agreement were selected. These 31 items were included in the final checklist and sorted into ten categories after a pilot study and adjustment of its content (Table 2). 

## Discussion

Maintaining high-quality CPR in the ED requires monitoring and feedback. Suboptimal CPR is reported among healthcare providers; there are also reports about the decline in the quality of CPR after transferring the care of victims from a pre-hospital setting to the ED (1). 

In this study, a group of specialists in the field of resuscitation and emergency medicine participated in two rounds of group discussions and one Delphi panel to reach a consensus on quality control of CPR in the ED. Eventually, 31 factors were identified and classified into ten categories. These 31 items were used to generate a checklist for quality control of CPR in the ED. Based on AHA guidelines, the rate of chest compression in CPR is 100-120/min (2, 8, 9). Since any pause in chest compression has a massive negative impact on the outcome of CPR, this factor plays a crucial role in performing high-quality CPRs (10).

Compression depth is an essential part of high-quality CPR and has been deemed related to patient survival (4, 11). 

While the recommended depth of chest compression in adults is 2 inches, Stiell et al. reported that there is no strict depth for chest compression (12). Furthermore, measuring the depth of chest compression needs equipment such as an accelerometer or impulse-radio ultra-wideband (IR-UWB) sensor (13). In a resource-limited ED, as is often the case in our country, these devices are not available, and the precise measurement of the depth of chest compression is not possible. Our participants emphasized the necessity for advanced devices. Nevertheless, our specialists arrived at a strong consensus about this item, and we included chest compression depth in our checklist. 

Cha et al., in 2015, reported that CPR for less than 20 min significantly decreases the survival of patients with cardiac arrest (14). There are many factors that impact the duration of CPR, including patients’ age, past medical history, prognosis of the current illness, and time of arrest (15). We could not identify any specific duration for CPR in our study.

We did not define different weights for our items. However, these items have different values, and we need to undertake more research to define a weight for each item. Moreover, there are other factors that impact the quality of medical care in ED, including CPR, such as overcrowding, lack of resources, and burnout and occupational stress among personnel (6). In order to maintain high-quality CPR, all these factors must be considered and resolved. If survival rates and neurologic outcomes in cardiac arrests in EDs are to be improved, it is crucial that ED staff understand all aspects of high-quality CPR. In conclusion, our study provides a checklist for monitoring the quality of CPR in the ED, but it is still necessary to include other factors related to the ED environment in this checklist.

## Conclusion:

Our study provides a checklist for monitoring the quality of CPR in the ED, but it is still necessary to include other factors related to the ED environment on this checklist.
